# Does physical and social neighborhood environment matter for two-year changes in functional abilities and cognitive function in the oldest old?

**DOI:** 10.1093/geronb/gbaf182

**Published:** 2025-09-23

**Authors:** Jaroslava Zimmermann, Gizem Hülür

**Affiliations:** Cologne Center for Ethics, Rights, Economics and Social Sciences of Health, University of Cologne, Cologne, Germany; Department of Psychology, University of Bonn, Bonn, Germany

**Keywords:** Activities of daily living, Cognition, Living conditions, Longitudinal change, Very old age

## Abstract

**Objectives:**

While favorable physical neighborhood environments have been shown to benefit functional abilities (FA) and cognitive function (CF) in older adults, evidence on social aspects remains inconclusive. This study aimed to examine the role of both physical (quality, infrastructure, walkability) and social (place attachment, social cohesion) neighborhood characteristics for levels/changes in FA and CF among the oldest old, who were often underrepresented in previous research. Additionally, we examined whether place attachment and social cohesion mediate the associations between physical neighborhood characteristics and FA and CF outcomes.

**Methods:**

We used data from the population-based NRW80+ survey, including two waves collected in 2017–2018 and 2019–2020. NRW80+ included the population aged 80 years or older with primary residence in North Rhine-Westphalia (Germany). Based on the structural equation framework, we estimated latent difference score models to examine levels/changes in FA (*n *= 840) and CF (*n *= 797) and to test mediation effects.

**Results:**

Higher walkability was related to better baseline FA, and an improvement in walkability ratings was associated with less FA decline. Neighborhood quality and infrastructure were not related to FA or CF. Higher social cohesion was associated with less CF decline. No mediation effects through place attachment and social cohesion were identified.

**Discussion:**

Our findings indicate that walkable neighborhood environments may help maintain FA, while socially cohesive neighborhoods may buffer against CF decline in the oldest old. The absence of mediation effects suggests that physical and social aspects of the neighborhood may influence FA and CF through independent pathways.

## Background

In the context of ongoing demographic change, the proportion of the oldest old (referring here to the population aged 80 years and older) is increasing rapidly. In Germany, the oldest old belong to the fastest growing age group, which is expected to rise from recently 7% to about 14% in the next decades (Statistisches Bundesamt, 2023). As a result of aging-associated loss of physiological reserves, the oldest age is frequently characterized by increased frailty and health limitations (Baltes & Smith, 2003). The World Health Organization (2015) identifies functional abilities (FA)—the capacity to perform activities of daily living—to emerge from the interaction between individual resources and environmental conditions. Although closely linked to FA, cognitive function (CF)—referring to the mental capacity to manage complex everyday tasks (e.g., memory, attention, or information processing)—can follow different trajectories in older age (Raimo et al., 2024).

## Neighborhood environment and aging process

Building on well-established theoretical perspectives in environmental gerontology, FA and CF can be understood as key outcomes in the aging process, shaped particularly by the physical and social environment (cf. Bronfenbrenner, 1979; Lawton & Nahemow, 1973; Wahl et al., 2012). With increasing age and health limitations, daily activities tend to concentrate within the immediate residential environment or neighborhood (Lawton, 1982). The empirical evidence confirms that various characteristics of the physical neighborhood environment (characteristics of the built and natural environment) are associated with FA (Molaei et al., 2024; Rachele et al., 2019) and CF (Chen et al., 2022; Da et al., 2025; Song et al., 2024; Sullivan et al., 2024) in older age. The evidence consistently shows that older adults living in neighborhoods with poor infrastructure (e.g., limited access to local amenities, low land use diversity), low environmental quality (e.g., physical disorder, lack of green spaces), or low walkability experience more limitations in FA (Molaei et al., 2024; Rachele et al., 2019) and worse CF (Chen et al., 2022; Da et al., 2025; Song et al., 2024; Sullivan et al., 2024). Compared to physical neighborhood characteristics, fewer studies focused on the impact of social neighborhood environment (social bonds among neighbors, social participation, sense of belonging) on FA (Molaei et al., 2024) and CF (Besser et al., 2017; Sullivan et al., 2024). Results, predominantly from cross-sectional studies, suggest a positive association between a more favorable social neighborhood environment (i.e., high neighborhood trust) on FA (Molaei et al., 2024) and CF (Sullivan et al., 2024). However, the systematic review by Besser et al. (2017) reported mixed results regarding the link between social relationships within neighborhoods and CF. The scoping review by Sullivan et al. (2024), which focused on minoritized population groups in the United States, highlighted cultural differences in the role of social cohesion (neighborhood trust) for cognitive outcomes, suggesting that its effects may be shaped by broader social and historical contexts.

In line with collective efficacy theory, Sampson et al. (2002, 1997) argued that deteriorated physical environments, including signs of disorder or poor infrastructure, may discourage residents from engaging with their neighborhoods. This withdrawal undermines opportunities for social interaction among neighbors and hinders the development of trust or informal support networks (social cohesion). Adverse neighborhood environments can contribute to the accumulation of psychological distress over time (Aneshensel et al., 2016), which has been shown to negatively affect aging outcomes, such as FA and CF (D’Amico et al., 2020; Guidi et al., 2021). On the other hand, a favorable physical environment may be beneficial for the oldest old to develop a strong sense of belonging, which may be beneficial for aging outcomes (Chaudhury & Oswald, 2019; Wahl et al., 2012). Belonging is defined as emotional and cognitive connectedness to one’s immediate environment, encompassing feelings of attachment, familiarity, and place identity.

## Physical and social neighborhood environment in relation to FA and CF

Few studies have examined the joint influence of physical and social neighborhood environments on FA (Liu et al., 2023; Millar, 2020; Qin et al., 2021) and CF in older adults (Farmer et al., 2024; Lee & Waite, 2018; Sharifian et al., 2020). However, none of these studies have investigated whether social environment characteristics mediate the effects of physical neighborhood conditions on FA and CF. Most findings confirmed that favorable physical and social neighborhood conditions are associated with better FA (Liu et al., 2023; Qin et al., 2021) and CF (Farmer et al., 2024; Lee & Waite, 2018). Nevertheless, findings regarding the longitudinal effects of neighborhood environment on FA (Liu et al., 2023; Qin et al., 2021) and CF (Sharifian et al., 2020) are inconclusive.

More specifically, a longitudinal study from Hong Kong found that higher accessibility to neighborhood public open spaces (e.g., shaded areas, benches) and higher levels of social interaction among space users were associated with higher independence in instrumental activities of daily living (IADL) at baseline and a slower decline over 4 years (Liu et al., 2023). Using nationally representative cross-sectional U.S. data, Millar (2020) reported that neighborhood physical disorder (based on interviewer-rated cleanliness and presence of vacant buildings) was negatively associated with physical function (score including walking speed, muscle strength, and balance). However, the effect of social cohesion (e.g., perceived neighborhood trust or willingness to help) became non-significant after adjusting for sociodemographic and health characteristics. Qin et al. (2021), analyzing longitudinal data over 8 years from the same cohort, found that neighborhood physical disorder also predicted the occurrence of IADL limitations. In contrast to Millar (2020), increases in social cohesion in the previous wave were found to reduce the rate of limitations and to lower the risk of new limitations in both basic activities of daily living and IADL in the subsequent wave.

With regard to CF, Sharifian et al. (2020), using nationally representative data from the Health and Retirement Study, found that higher neighborhood physical disorder (based on perceived safety and cleanliness) was associated with poorer episodic memory and verbal fluency at baseline. Although neighborhood social cohesion (measured through perceived trust and sense of belonging) had no direct effect on CF, it showed an indirect positive impact through reduced psychosocial distress (e.g., anxiety), contributing to higher CF and a slower decline in verbal fluency over 4 years. Similarly, a cross-sectional study using Health and Retirement Study data on the older Black population reported that higher neighborhood physical disorder was associated with lower CF (based on mental processing speed, episodic memory, and working memory), and high (but not moderate) levels of social discohesion were also linked to lower CF in this group (Farmer et al., 2024). Another cross-sectional study based on nationally representative U.S. data demonstrated that older adults living in neighborhoods with higher levels of building neglect and lower social cohesion had lower CF (based on orientation, executive function, visuospatial skills, memory, language, and memory) (Lee & Waite, 2018).

## The present study

Although ample research demonstrates the relevance of both physical and social neighborhood environments for FA and CF in older age, findings from longitudinal studies are inconsistent depending on measures, sample size, target population, and region (Liu et al., 2023; Qin et al., 2021; Sharifian et al., 2020). Furthermore, most studies were conducted in North American and Asian countries. Since the effect of neighborhood environment on aging outcomes may differ across geographic locations due to differences in cultural contexts, available public resources, and health and welfare systems (Jiang et al., 2020; Ribeiro et al., 2018), previous findings might not be generalizable to the European context. Additionally, previous research has mostly focused on “young-old” adults (approximately 60–80 years old) who are—in contrast to their “oldest-old” counterparts—rather healthy and active (Baltes & Smith, 2003). Given that individual competencies and resources decrease as people age, one can expect that the oldest old might be particularly affected by their neighborhood environment (Lawton & Nahemow, 1973). However, this age group is often underrepresented or excluded in large-scale surveys due to considerable health limitations or residence in institutional settings (Kelfve, 2019). This may lead to an underestimation of the environmental impact on aging outcomes.

**Figure 1.**
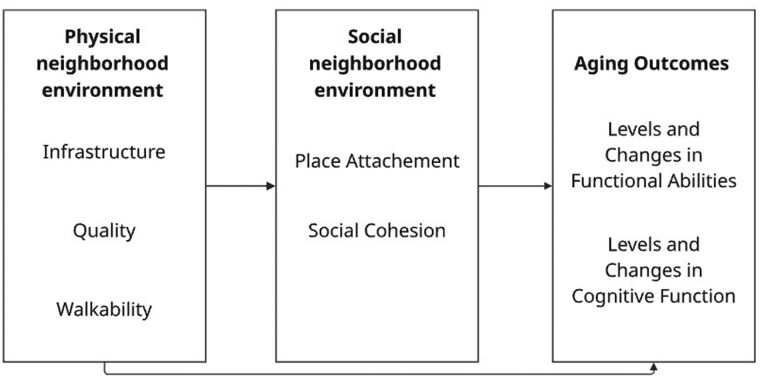
Conceptual Framework. Potential pathways underlying associations between physical neighborhood environment, social neighborhood environment, and aging outcomes in the oldest old. The solid-lined arrows represent explicitly tested associations.

Therefore, the present study aimed, first, to examine the role of physical and social neighborhood environment for levels and changes in FA and CF using representative longitudinal data of the oldest old living in the most populous federal state in Germany. As depicted in Figure 1, we hypothesized that favorable physical and social neighborhood environments would be associated with higher levels of FA and CF and with slower decline over time. The second aim was to examine whether the association between physical neighborhood characteristics and levels/changes in FA and CF is mediated by social neighborhood environment. Based on our conceptual framework (see Figure 1), we assumed that poor physical neighborhood environments may inhibit the development of social relationships among neighbors (such as social cohesion), thereby increasing psychological distress and, consequently, contributing to lower levels of FA and CF as well as a faster decline. On the other hand, favorable physical neighborhood environments can foster a strong sense of belonging to the neighborhood, which can, in turn, be related to more favorable levels/changes in FA and CF in the oldest old.

## Method

### Study sample

We utilized data from the population-based panel study “Quality of Life and Well-Being of the Very Old in North Rhine-Westphalia” (NRW80+), including the population aged 80 years and older with the primary residence in North Rhine-Westphalia (Germany). The study sample included individuals living in private households and nursing care facilities. The sampling procedure of NRW80+ was based on a two-stage approach. First, 94 municipalities were randomly selected, followed by the random selection of 8,040 individuals. In case the target person was unable to participate due to considerable health limitations, the interview was conducted with a proxy. NRW80+ is based on two waves of data collection using computer-assisted personal interviews. The data of Wave 1 (W1) were collected between 2017 and 2018 with 1,863 participants. The Wave 2 (W2) was conducted between 2019 and 2021 and consisted of the follow-up survey (*n *= 912) and the baseline survey of a refreshment sample (*n *= 950). The data collection of the follow-up survey was completed in February 2020, which was shortly before the incidence rate of COVID-19 began to accelerate in Germany. Due to the pandemic restrictions, the data of the refreshment sample were partly collected via written questionnaires. The study protocol of NRW80+ was approved by the ethical board of the Medical Faculty at the University of Cologne (Protocol No.:17-169). More details about the study approach of NRW80+ are published elsewhere (Hansen et al., 2021).

The current study used data exclusively from individuals who participated in both waves and completed at least one task at each wave in the assessment of CF or answered at least one item of the scale measuring FA at each wave. Interviews with proxies, as well as those with a change of informant between W1 and W2, were excluded due to the use of a different assessment tool (CF) and measurement invariance (FA) identified between interviews with target persons and proxies (Kaspar et al., 2024). The final analytic sample included 840 participants for the FA outcome and 797 participants for the CF outcome. In all analyses, longitudinal sampling weights were applied to account for the complex study design and to correct for potential biases in panel recruitment, particularly due to mortality or unwillingness to participate in W2. These were computed based on baseline risk factors that predicted dropout in W2 (e.g., higher age, female sex, low socioeconomic status, lower cognitive and physical function, or living in a nursing home). The inverse probabilities of these factors were used as weights to adjust for panel selectivity (Wagner & Zank, 2022).

### Measures

#### Outcome variables

To assess *cognitive function*, the DemTect screening tool (Kalbe et al., 2004) was used in both waves. The DemTect consists of five tasks, including immediate recall, number transcoding, word fluency, digit span backwards, and delayed recall. The immediate recall task required participants to remember as many words as possible (in no particular order) from a list of 10 words read by the interviewer. This task was repeated once, using the same list of words read out again and asking the participants to remember as many words as possible. The task score ranged between 0 and 20. The number transcoding task consisted of converting sequences of digits into number words and vice versa. The score ranged from 0 to 4. The word fluency task required participants to name as many items as possible that can be purchased in a supermarket within 60 s. The score corresponded to the number of correctly named items. The digit span backwards task involved recalling the sequence of digits in reverse order after each sequence. Each sequence included 2–6 single-digit numbers. The score varied from 0 to 6. The delayed recall task required participants to remember as many words as possible from the word list of the first task (immediate recall). The score ranged between 0 and 10. As in our previous work (Hülür & Zimmermann, 2025), we z-standardized all scores using the weighted sample mean and the *SD* at the time of W1.

*Functional abilities* were measured in both waves by self-reported independence in IADL based on Lawton and Brody (1969). Participants reported the amount of help they needed to perform the following activities: using the telephone, organizing routes outside the walking range (e.g., taking a bus or taxi), buying food and clothes, preparing meals, doing housework, taking medication, and handling finances. The response options were 0 = “not possible without help,” 1 = “some help needed,” or 2 = “no help needed.”

#### Independent variables

To operationalize *physical neighborhood environment*, we used two measures assessed by interviewers (quality of neighborhood physical environment and neighborhood age-friendly infrastructure) and self-rated walkability collected in both waves. *Neighborhood quality* was defined as the availability of warm materials (e.g., wood or brick), comfortable benches, diverse vegetation, places offering shade, pleasantly designed boundaries (e.g., fences or hedges), or similar features. Interviewers had four response options ranging from 1 = “not attractive at all” to 4 = “very attractive.” *Neighborhood infrastructure* was defined as the availability of benches, pedestrian sidewalks, handrails, outdoor lighting, or similar features. Interviewer ratings ranged from 1 = “not functional at all” to 4 = “very functional.” Neighborhood quality showed a slight improvement, while infrastructure significantly deteriorated over time. However, we did not consider these differences to be changes in the neighborhood environment, as they may result from different interviewers at W1 and W2. To enhance the objectivity of ratings, we used mean interviewer ratings from W1 and W2 for each characteristic. *Walkability* was measured by one item asking participants about the suitability of the external living environment for walking, using a wheelchair, or managing things. The response options ranged from 1 = “not suitable at all” to 4 = “very suitable.” Walkability ratings significantly decreased over time. In addition to baseline walkability, the difference in ratings between W2 and W1 was considered. Negative values indicated deterioration, and positive values reflected an improvement in the rating over time.

*Social neighborhood environment* was assessed using two items collected in both waves: place attachment and social cohesion. To assess *place attachment*, participants were asked how closely they feel connected to the outdoor living environment, with response options ranging from 1 = “not close at all” to 4 = “very close.” *Social cohesion* was operationalized by asking participants about trusting their neighbors (inside and outside the residential building). The response options varied from 1 = “strongly disagree” to 5 = “strongly agree.” While place attachment showed a significant increase, social cohesion decreased over time. For both measures, the difference in ratings between W2 and W1 was considered in addition to the baseline ratings. Negative values indicated deterioration, while positive values indicated an improvement in the rating over time.

#### Control variables

The following baseline variables were included as controls: *age* (in years), *sex* (men/women), *partnership status* (not married or no partner/married or partnered), *living alone* (no/yes), *length of residency* in current apartment/facility (in years), *living in a nursing care facility* (no/yes), *physical activity* (monthly or less/weekly or more), and *individual socioeconomic status* determined by the participant’s last profession before retirement based on the International Standard Classification of Occupation (Ganzeboom, 2010) ranging from 16 (e.g., helpers or cleaners) to 90 (judges).

### Statistical analyses

The analyses were conducted in several steps. First, we conducted descriptive (Table 1) and correlational analyses of included variables (see online supplementary material, Supplementary Tables 1 and 2).

**Table 1.** Descriptive statistics at baseline according to the aging outcome in the oldest old.

Variable	Functional abilities (*n *= 840)		Cognitive function (*n *= 797)	
	% or *M*	*SD*	% or *M*	*SD*
**Using telephone W1^a^**				
** Not possible without help**	1.500			
** Some help needed**	2.600			
**Organizing routes W1^a^**				
** Not possible without help**	18.800			
** Some help needed**	10.800			
**Buying food and clothes W1^a^**				
** Not possible without help**	22.000			
** Some help needed**	12.100			
**Preparing meals W1^a^**				
** Not possible without help**	17.100			
** Some help needed**	11.400			
**Doing housework W1^a^**				
** Not possible without help**	24.600			
** Some help needed**	30.100			
**Taking medication W1^a^**				
** Not possible without help**	13.600			
** Some help needed**	6.600			
**Handling finances W1^a^**				
** Not possible without help**	19.000			
** Some help needed**	12.800			
**Immediate recall W1**			2.476	0.952
**Number transcoding W1**			2.355	0.884
**Word fluency W1**			3.326	1.092
**Digit span backwards W1**			2.654	0.630
**Delayed recall W1**			3.505	1.970
**Sex W1 (female)**	62.500		62.300	
**Age W1 (in years)**	85.395	4.032	85.269	4.028
**Socioeconomic status W1**	42.033	20.931	42.553	21.201
**Length of residency W1 (in years)**	38.703	26.975	38.951	26.973
**Married/partnered W1**	41.300		41.900	
**Living alone W1**	47.700		48.500	
**Living in a nursing facility W1**	11.100		10.700	
**At least weekly physically active W1**	84.900		85.400	
**Neighborhood infrastructure^b^**	2.016	0.645	2.019	0.646
**Neighborhood quality^b^**	2.083	0.644	2.090	0.650
**Walkability W1**	1.803	0.979	1.827	0.982
**Walkability difference^c^**	−0.099	1.022	−0.112	1.040
**Place attachment W1**	2.186	0.958	2.204	0.952
**Place attachment difference^c^**	0.009	1.078	0.017	1.087
**Social cohesion W1**	3.213	1.031	3.243	1.006
**Social cohesion difference^c^**	−0.046	0.975	−0.047	0.945

*Note.* Panel weighted data.

a The category “No help needed” was used as a reference.

b Mean value based on rating of Waves 1 and 2. Higher values indicate more favorable conditions.

c Computed as a difference in ratings between Wave 2 and 1. Negative values indicate a deterioration, and positive values indicate an improvement in rating.

Second, we specified baseline measurement models for FA and CF using structural equation modeling. A single factor was specified for FA and CF each, reflecting individual differences in FA and CF based on the shared variance of the single indicators.

Third, we tested mean level differences/stability in constructs determining FA and CF over time. To be able to compare the mean scores over time, strong measurement invariance is required (Widaman et al., 2010). Measurement invariance was tested by linking the single indicators of FA and CF with latent variables at each time of measurement (W1 and W2) and by comparing fit indices of configural (free factor loadings across time), metric (equality of factor loadings across time), and scalar (equality of factor loadings and intercepts across time) models (Widaman et al., 2010). In the case of CF, we applied the identical approach as in our earlier publication (Hülür & Zimmermann, 2025). As all CF indicators were continuous, the maximum likelihood estimator with robust standard errors was used in the models with the CF outcome. The model fit was evaluated using the Comparative Fit Index (CFI), the Root Mean Square Error of Approximation (RMSEA), and the Standardized Root Mean Square Residual (SRMR). Models with values of RMSEA and SRMR lower than 0.08 and CFI higher than 0.95 were considered as good model fit, and CFI higher than 0.90 as acceptable model fit (Hu & Bentler, 1999). Since FA indicators were measured on an ordinal scale, the weighted least squares mean and variance adjusted estimator with probit link was used in models with FA outcome. In case of FA, RMSEA and CFI were used to evaluate model fit, as they were identified as suitable fit indices in models using the weighted least squares mean and variance adjusted estimator (Yu, 2002).

Fourth, separate latent change score models were specified for FA and CF. The latent variables CF at W1 and FA at W1 were estimated based on indicators measured in W1. Changes in outcomes between W1 and W2 were captured by a latent difference variable specified as a difference between W2 and W1 (Geiser, 2021).

Fifth, characteristics of the physical neighborhood environment (quality, infrastructure, and walkability) were added as predictors of FA and CF level/change in separate models. Self-reported walkability was assessed in both waves. Ratings at W1 were included as predictors of baseline levels of FA and CF, while both the W1 ratings and their change over time (defined as the difference between W2 and W1 ratings) were included as predictors of FA and CF changes. The models were adjusted for age, sex, partnership status, living alone, length of residency, living in a nursing care facility, physical activity, and individual socioeconomic status.

Sixth, indicators of social neighborhood environment (place attachment and social cohesion) were additionally included as predictors of FA and CF level/change. As both neighborhood indicators were collected in both waves, ratings reported in W1 were included as predictors of FA and CF levels. Ratings at W1 and their change over time (defined as the difference between rating at W2 and rating at W1) were included as predictors of FA and CF changes. The results of the original (non-imputed) dataset are presented in online supplementary material, Supplementary Tables 6 (FA) and Table 7 (CF).

Seventh, we analyzed whether the social neighborhood environment at W1 mediated the link between physical neighborhood characteristics (specified as exogenous variables) and FA and CF level/change (specified as endogenous variables). The mediation analyses were estimated based on the latent change score models described in previous steps for each outcome. All mediators were allowed to correlate with each other. This also applied to the exogenous variables. All control variables were included as confounders of the level/change in both outcomes and of all mediators. The size of the mediation effect (specific indirect effect) of each mediator was estimated as the percentage of change in the regression coefficients (effect of specific exogenous variable on outcome) when the mediators were included in the model (Ditlevsen et al., 2005).

All analyses were performed using Mplus 8.8 software (Muthén & Muthén, 1998–2017). Statistical significance was set at *p *< .05. Incomplete data were assumed to be missing at random. Multiple imputation was used to replace missing values of independent and control variables. We generated 20 datasets separately for each sample using all variables included in the analyses. The highest missing rate was identified for social cohesion at W2 (about 3.5% in both samples).

## Results

Table 1 provides descriptive statistics of all included variables, and their intercorrelations are reported in online supplementary material, Supplementary Table 1 (FA) and Supplementary Table 2 (CF).

In a second step, separate confirmatory models with a general factor were specified for FA and CF at W1. Both single-factor models, FA (CFI = 0.993; RMSEA = 0.077) and CF (CFI = 0.977; RMSEA = 0.048; SRMR = 0.029), showed good model fit. In the CF model, a residual covariance was specified between immediate and delayed recall.

Third, we tested the measurement invariance of FA and CF between W1 and W2 in separate models. For both outcomes, the scalar models showed good model fit, and fit indices showed no significant deterioration after restricting factor loadings and intercepts. In other words, the assumption of strong measurement invariance could be confirmed. Results of measurement invariance analyses are presented in online supplementary material, Supplementary Table 3.

The fourth step included fitting separate latent change score models to analyze individual differences in W1 and changes after 2 years in FA and CF. The model for FA (see online supplementary material, Supplementary Table 4) and for the CF outcome (see online supplementary material, Supplementary Table 5) showed good model fit. Factor loadings of both outcomes were significantly different from zero (all *p* < .001), and participants showed a decline in FA and CF over time. In both outcomes, participants significantly differed in the extent of decline.

Fifth, characteristics of the physical environment (quality, infrastructure, walkability) were added as predictors of FA (Model 1 in Table 2) and CF (Model 1 in Table 3) level/change. In both models, neighborhood quality and infrastructure were not associated with levels/changes in FA and CF. Participants who perceived their neighborhood as more walkable showed a higher FA level (*b *= 0.355; *SE *= 0.126; *p =* .005; β = 0.150), and those who rated neighborhood walkability in W2 better compared to W1 showed less FA decline over time (*b *= 0.266; *SE *= 0.088; *p =* .003; β  = 0.259). None of the walkability measures were related to CF levels/changes.

**Table 2.** Results from latent change score models predicting level and change in functional abilities in the oldest old.

Variable	Functional abilities: Model 1						Functional abilities: Model 2					
	Level			Change			Level			Change		
	Est	*SE*	Std	Est	*SE*	Std	Est	*SE*	Std	Est	*SE*	Std
**Sex W1 (female)**	**−0.427**	0.179	−0.089	0.268	0.142	0.124	**−0.417**	0.183	−0.087	0.261	0.148	0.121
**Age W1**	**−0.130**	0.031	−0.225	−0.006	0.014	−0.024	**−0.128**	0.032	−0.223	−0.007	0.014	−0.028
**Socioeconomic status W1**	**0.014**	0.005	0.122	0.000	0.003	−0.008	**0.014**	0.005	0.129	0.000	0.003	−0.002
**Length of residency W1**	0.007	0.004	0.076	0.003	0.002	0.074	0.004	0.004	0.050	0.004	0.003	0.109
**Married/partnered W1**	**1.411**	0.376	0.299	−0.396	0.218	−0.186	**1.395**	0.383	0.297	−0.351	0.223	−0.166
**Living alone W1**	**1.422**	0.341	0.306	−0.244	0.165	−0.116	**1.391**	0.343	0.300	−0.195	0.177	−0.093
**Living in a nursing facility W1**	**−1.443**	0.450	−0.196	0.531	0.313	0.159	**−1.451**	0.441	−0.197	0.554	0.307	0.167
**At least weekly physically active W1**	**1.333**	0.325	0.205	**−0.386**	0.167	−0.131	**1.312**	0.321	0.203	**−0.384**	0.160	−0.132
**Neighborhood infrastructure^a^**	0.023	0.186	0.006	−0.097	0.127	−0.060	0.059	0.187	0.016	−0.118	0.123	−0.073
**Neighborhood quality^a^**	0.145	0.174	0.040	0.005	0.112	0.003	0.077	0.182	0.021	0.015	0.115	0.009
**Walkability W1**	**0.355**	0.126	0.150	−0.047	0.080	−0.043	**0.327**	0.128	0.138	−0.032	0.079	−0.030
**Walkability difference^b^**				**0.266**	0.088	0.259				**0.259**	0.094	0.254
**Place attachment W1**							0.140	0.136	0.058	−0.120	0.114	−0.110
**Social cohesion W1**							0.066	0.110	0.030	0.066	0.084	0.066
**Place attachment difference^b^**										0.004	0.072	0.004
**Social cohesion difference^b^**										0.128	0.089	0.120
**R^2^**	0.444			0.196			0.449			0.211		
**RMSEA**	0.035						0.029					
**CFI**	0.971						0.974					

Note. *N* = 840. Panel-weighted data. Est = Unstandardized Estimate; *SE* = Standard Error; Std = Standardized Estimate; W1 = Wave 1; RMSEA = Root Mean Square Error of Approximation; CFI = Comparative Fit Index. Parameters which were statistically significant at the *p* < .05 level are shown in bold font.

a Mean value based on ratings of Waves 1 and 2. Higher values indicate more favorable conditions.

b Computed as a difference in ratings between Waves 2 and 1. Negative values indicate a deterioration, and positive values indicate an improvement in rating.

**Table 3.** Results from latent change score models predicting level and change in cognitive function in the oldest old.

Variable	Cognitive function: Model 1						Cognitive function: Model 2					
	Level			Change			Level			Change		
	Est	SE	Std	Est	SE	Std	Est	SE	Std	Est	SE	Std
**Sex W1 (female)**	**0.163**	0.065	0.121	0.033	0.041	0.050	**0.160**	0.066	0.119	0.028	0.042	0.042
**Age W1**	**−0.013**	0.003	−0.081	**−0.004**	0.002	−0.049	**−0.013**	0.003	−0.083	**−0.005**	0.002	−0.064
**Socioeconomic status W1**	**0.009**	0.002	0.285	0.000	0.001	−0.013	**0.009**	0.002	0.286	0.000	0.001	−0.014
**Length of residency W1**	**0.004**	0.001	0.156	0.002	0.001	0.152	**0.003**	0.001	0.135	0.002	0.001	0.153
**Married/partnered W1**	**0.317**	0.125	0.239	**0.152**	0.077	0.233	**0.313**	0.123	0.236	0.140	0.078	0.215
**Living alone W1**	**0.316**	0.116	0.242	0.095	0.090	0.148	**0.310**	0.113	0.237	0.098	0.091	0.152
**Living in a nursing facility W1**	**−0.476**	0.195	−0.225	0.271	0.151	0.261	**−0.464**	0.194	−0.219	0.267	0.151	0.257
**At least weekly physically active W1**	0.205	0.110	0.111	0.027	0.079	0.029	0.202	0.108	0.109	0.022	0.078	0.024
**Neighborhood infrastructure^a^**	0.091	0.064	0.090	−0.015	0.058	−0.030	0.100	0.065	0.099	−0.021	0.059	−0.043
**Neighborhood quality^a^**	−0.011	0.060	−0.011	−0.001	0.050	−0.002	−0.024	0.065	−0.023	−0.018	0.051	−0.037
**Walkability W1**	−0.030	0.031	−0.045	0.011	0.028	0.034	−0.037	0.032	−0.055	0.005	0.030	0.016
**Walkability difference^b^**				−0.001	0.028	−0.004				−0.004	0.027	−0.013
**Place attachment W1**							0.053	0.040	0.077	−0.035	0.029	−0.104
**Social cohesion W1**							−0.014	0.028	−0.022	**0.078**	0.031	0.245
**Place attachment difference^b^**										−0.012	0.021	−0.041
**Social cohesion difference^b^**										0.026	0.028	0.076
**R^2^**	0.325			0.054			0.330			0.094		
**RMSEA**	0.032						0.027					
**CFI**	0.936						0.942					
**SRMR**	0.044						0.041					

*Note*. *N* = 797. Panel-weighted data. Est = unstandardized estimate; *SE* = standard error; Std = standardized estimate; W1 = Wave 1; RMSEA = Root Mean Square Error of Approximation; CFI = Comparative Fit Index; SRMR = Standardized Root Mean Square Residual. Z-standardized values based on weighted means and *SD*s at baseline were used for manifest variables. Parameters that were statistically significant at the *p* < .05 level are shown in bold font.

a Mean value based on ratings of Waves 1 and 2. Higher values indicate more favorable conditions.

b Computed as a difference in ratings between Waves 2 and 1. Negative values indicate a deterioration, and positive values indicate an improvement in rating.

Sixth, indicators of social environment (place attachment, social cohesion) were included in addition to physical characteristics as predictors of FA (Model 2 in Table 2) and CF (Model 2 in Table 3) level/change. Indicators of social neighborhood environment were not associated with levels/changes in FA and baseline CF. The effect of walkability at W1 on FA level (*b *= 0.327; *SE *= 0.128; *p =* .011; β = 0.138), as well as the effect of walkability change on FA decline (*b *= 0.259; *SE *= 0.094; *p =* .006; β = 0.254), slightly decreased but remained statistically significant. None of the physical and social neighborhood characteristics were related to CF levels. Participants who reported higher social cohesion at W1 showed less CF decline over time (*b *= 0.078; *SE *= 0.031; *p =* .013; β = 0.245). No association was found between changes in social cohesion and CF decline.

**Figure 2.**
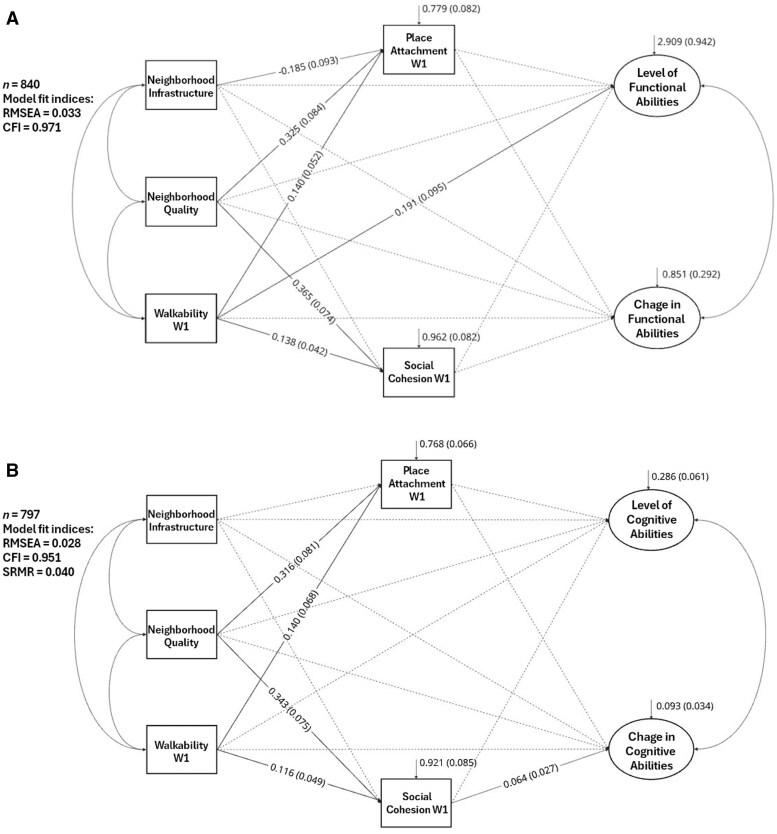
Mediational pathways from physical neighborhood characteristics to (A) functional abilities and (B) cognitive function in the oldest old. Panel-weighted data. W1 = Wave 1; RMSEA = Root Mean Square Error of the Approximation; CFI = Comparative Fit Index; SRMR = Standardized Root Mean Square Residual. The path analyses show associations between characteristics of physical neighborhood environment (infrastructure, quality, walkability), indicators of social neighborhood environment (place attachment, social cohesion), and level/change in functional abilities (Panel A) and cognitive function (Panel B) among the oldest old. For all mediators and endogenous variables, residual variances with standard errors in parentheses are presented. The coefficients presented are unstandardized estimates with standard errors in parentheses. The solid-lines indicate statistically significant associations at the *p* < .05 level. The dashed-lines indicate associations not significantly different from zero.

Lastly, we examined the mediation effects of social neighborhood environment at W1 on the link between physical characteristics of neighborhoods and FA (Figure 2A) and CF (Figure 2B) level/change. None of the indicators of social neighborhood environment mediated the associations between physical neighborhood characteristics and FA or CF level/change. Supplementary Tables 8 and 9 (see online supplementary material) provide further details on the findings of the mediation analyses for levels/changes in FA and CF, respectively.

## Discussion

The current study contributes to the evidence on the role of neighborhood environments for disparities in aging outcomes by focusing on the oldest old, a population group often underrepresented in previous research. Partially supporting our hypotheses, our results predominantly indicated that more favorable physical and social neighborhood environments were associated with better aging outcomes at baseline and with less decline over the 2-year period. However, the associations differed between FA and CF outcomes. With respect to FA, higher baseline ratings of perceived walkability—an indicator of physical neighborhood environment—were related to higher FA at baseline. Moreover, improvements in walkability ratings over time were associated with less decline in FA. Interviewer-rated physical neighborhood characteristics (quality and age-friendly infrastructure) as well as indicators of the social neighborhood environment (place attachment and social cohesion) were unrelated to FA levels/changes. None of the physical or social neighborhood characteristics were associated with baseline CF. However, higher levels of baseline social cohesion were linked to a slower decline in CF over time. Physical neighborhood characteristics and place attachment showed no association with changes in CF. Our analyses did not support the hypothesis, derived from collective efficacy theory (Sampson et al., 1997, 2002), that the social neighborhood environment mediates the association between physical neighborhood environment and aging outcomes.

Consistent with earlier research (Molaei et al., 2024), we found that living in a more walkable neighborhood was associated with better FA at baseline. Although the current study used a subjective measure, our results are in line with previous studies using exclusively objective walkability assessments (Fogal et al., 2019; Koohsari et al., 2020; Loh et al., 2019). The evidence shows that living in neighborhoods that are subjectively and objectively more walkable promotes physical activity and walking in older age (Barnett et al., 2017). Physical activity is one of the key predictors of FA (Roberts et al., 2017). Our analysis confirmed that the oldest old who engaged in physical activity at least once a week reported better FA at baseline than those who were less active. Thus, we expect that individuals who perceived their neighborhood as walkable were also more physically active, which might have positively affected their FA. It is also possible that individuals with better FA view their neighborhood as more walkable because they experience fewer barriers. Additionally, the oldest old individuals with more limitations in FA may be particularly vulnerable to barrier-rich neighborhood environments (Lawton, 1982), which can lead to increased psychological distress (Aneshensel et al., 2016) and, consequently, to further deterioration of their FA (cf. Guidi et al., 2021).

While no other longitudinal study examining this relationship could be identified (Molaei et al., 2024), our findings suggest that improvements in walkability ratings over the 2-year period were linked to less decline in FA. There might be several explanations. It is possible that adjustments to the residential environment to improve neighborhood walkability could have led to increased physical activity and consequently to less FA decline among the oldest old. Another explanation can be based on the work of Wahl and Oswald (e.g., Chaudhury & Oswald, 2019; Wahl et al., 2012). The authors demonstrated that individuals are able to adapt to adverse environmental conditions through proactive strategies to maintain control over their environment, even in very old age. For example, the oldest old may have started using a walking aid due to mobility limitations. Consequently, their perception of neighborhood walkability may have improved, resulting in increased physical activity and less functional decline over time.

Our finding that social cohesion at baseline was not associated with CF levels is inconsistent with findings of most previous studies (Farmer et al., 2024; Lee & Waite, 2018; Tang et al., 2020; Zaheed et al., 2019). However, we found that higher baseline social cohesion was related to less CF decline over time, whereas previous longitudinal studies did not identify such associations (Sharifian et al., 2020; Tang et al., 2020). These inconsistencies might be attributed to cultural and/or age-related differences, as prior studies were conducted in the United States and included a population aged about 50 years and older. Although social cohesion did not predict baseline CF in our sample, it was a relevant predictor of changes in CF. Operationalized as perceived neighborhood trust, social cohesion may be understood in terms of belonging as emotional and cognitive connections to the neighborhood, which was found to have a protective effect on aging outcomes in the oldest old (Chaudhury & Oswald, 2019; Wahl et al., 2012). Moreover, social relationships among neighbors can provide important resources that buffer against the adverse health consequences of acute or chronic stressors (e.g., illness, functional limitations) (cf. Thoits, 2011), thereby preventing cognitive decline (D’Amico et al., 2020).

Contrary to our expectation, place attachment was not associated with either FA or CF level/change in the oldest old. To the best of our knowledge, the current study examined this link for the first time in this age group. Although based on a younger population (aged 50 and older), our findings may align with results reported by Sharifian et al. (2020), who considered belonging to the neighborhood as one dimension of social cohesion. The authors showed that the positive effect of social cohesion on CF level/change was fully mediated by reduced psychological distress. While previous studies on the oldest old found stronger attachment to the neighborhood to be associated with decreased frailty risk (Zimmermann et al., 2021) and better self-rated health (Zimmermann, 2024), its influence on FA and CF may operate indirectly through individual psychological resources.

Furthermore, inconsistent with previous research (Da et al., 2025; Song et al., 2024), we found no association between walkability and CF level/change. While previous studies used objective measures, differential findings may be due to the use of a perceived walkability measure in the current study. Additionally, the absence of a direct association may point to the presence of indirect pathways, such as through physical activity (cf. Bherer et al., 2013), that were not captured in our analyses. The discrepancy in findings may also relate to the advanced age of participants; in the oldest old, sensory limitations such as impaired vision may restrict outdoor mobility. Thus, individuals may rarely leave their homes, regardless of how walkable they perceive their environment.

The lacking associations between neighborhood infrastructure or quality and the two aging outcomes contradict our hypotheses and the previous evidence (e.g., Molaei et al., 2024; Rachele et al., 2019; Song et al., 2024; Sullivan et al., 2024). These differences could arise from the use of interviewer ratings instead of objectively measured indicators of physical environment, which have been used more frequently in previous studies. On the other hand, subjective assessments of neighborhood infrastructure and quality might be more relevant for the FA and CF of the oldest old than interviewer-rated or objective characteristics, as they capture individual appraisals and emotional meaning, which become increasingly important mechanisms through which the environment influences aging outcomes in very late life (Chaudhury & Oswald, 2019; Wahl et al., 2012).

Similarly, our finding that social cohesion was not associated with FA in the oldest old is inconsistent with most previous studies (Molaei et al., 2024; Qin et al., 2021). These discrepancies may reflect age-related differences, as previous studies primarily included “young-old” adults, and none of them focused on the oldest old. The absence of an association might indicate that close social relationships (e.g., with family or friends) are more important social resources for maintaining FA in very old age than neighborhood social ties (cf. Thoits, 2011). In line with socioemotional selectivity theory (Carstensen, 2006), social networks tend to shrink with age, driven by prioritizing emotionally meaningful relationships. Our analysis confirmed that having a partner or being married was associated with better baseline FA.

Finally, the lack of a mediation effect through social neighborhood environment contrasts with the findings of previous studies based on the general population (Kress et al., 2020; Sampson et al., 1997, 2002). The oldest old are often underrepresented or entirely excluded from such large-scale population surveys, which limits the generalizability to this age group. Furthermore, the oldest old represent a selective population group due to their advanced age. Evidence indicates that individuals with lower socioeconomic status, who more often reside in socioeconomically deprived neighborhoods, are less likely to reach very old age (Mackenbach et al., 2016). Thus, the absence of mediation effects in our study may be attributable to survival bias. Alternatively, the association between the physical neighborhood environment and aging outcomes in the oldest age might be explained by other underlying mechanisms, such as close social relationships (cf. Carstensen, 2006; Thoits, 2011) or psychological distress (cf. Aneshensel et al., 2016), which were not accounted for in the present study.

### Limitations

Several limitations need to be considered. First, interviewer-reported and perceived neighborhood ratings were used to operationalize physical environment. Due to the lack of detailed information on interviewer assignments, we were not able to address potential interviewer bias. Future studies should include indicators from geographic information systems in addition to perceived measures. Second, further neighborhood characteristics that were not assessed within NRW80+ might considerably contribute to explaining differences in level and change of FA and CF in the oldest old (e.g., availability of green spaces, neighborhood socioeconomic deprivation, safety). Similarly, we were unable to fully account for neighborhood self-selection, for instance, based on unobserved individual residential preferences. However, we controlled for key individual characteristics (e.g., age, socioeconomic status, or partnership status) that are related to neighborhood choices (Besser et al., 2021). Third, since the available data included only two waves collected over a 2-year period, long-term health changes could not be investigated. Fourth, we were not able to examine the role of neighborhood environment for different cognitive domains (e.g., verbal fluency or processing speed) since NRW80+ involved only a comprehensive assessment of general cognition.

## Conclusions

The findings suggest that the role of physical and social neighborhood environment varies depending on the aging outcome in the oldest old. While walkable environments may support the maintenance of FA, socially cohesive neighborhoods could help protect against cognitive decline. These results emphasize the importance of treating physical and social neighborhood characteristics as distinct constructs with potentially independent effects on FA and CF. The lack of mediation effects suggests that these dimensions may operate through different mechanisms in very old age compared to younger age groups. Urban planning and community initiatives should acknowledge the distinct needs of the oldest old and actively include this group in both the design and implementation of age-friendly environments.

## Supplementary Material

gbaf182_Supplementary_Data

## Data Availability

Survey materials of NRW80+ and deidentified data sets for Waves 1 and 2 are available as scientific use files at the GESIS—Leibniz Institute for the Social Sciences data repository: https://doi.org/10.4232/1.13985. NRW80+ was preregistered (Clinical Trial Number: DRKS00011924).
