# Supplementary material for: Does physical and social neighborhood environment matter for two-year changes in functional abilities and cognitive function in the oldest old?
Source: J Gerontol B Psychol Sci Soc Sci. 2025 Sep 23;80(12):gbaf182. doi: 10.1093/geronb/gbaf182 (PMC12605762; doi:10.1093/geronb/gbaf182)
Supplement: gbaf182_Supplementary_Data [file gbaf182_supplementary_data.zip › JGSS suppl Zimmermann & Hülür.docx]

***The Journals of Gerontology, Series B: Psychological Sciences and Social Sciences* Supplementary Material: Zimmermann & Hülür. Does Physical and Social Neighborhood Environment Matter for Two-Year Changes in Functional Abilities and Cognitive Function in the Oldest Old?**

**Supplementary Table 1.** *Functional Abilities: Correlations of Included Variables*

| Variables ^a^ | 1 | 2 | 3 | 4 | 5 | 6 | 7 | 8 | 9 | 10 | 11 | 12 | 13 | 14 | 15 | 16 | 17 | 18 | 19 | 20 | 21 | 22 |
| --- | --- | --- | --- | --- | --- | --- | --- | --- | --- | --- | --- | --- | --- | --- | --- | --- | --- | --- | --- | --- | --- | --- |
| 1. Using telephone W1 | - |  |  |  |  |  |  |  |  |  |  |  |  |  |  |  |  |  |  |  |  |  |
| 2. Organizing routes W1 | 0.33 | - |  |  |  |  |  |  |  |  |  |  |  |  |  |  |  |  |  |  |  |  |
| 3. Buying food and clothes W1 | 0.31 | 0.82 | - |  |  |  |  |  |  |  |  |  |  |  |  |  |  |  |  |  |  |  |
| 4. Preparing meals W1 | 0.33 | 0.65 | 0.67 | - |  |  |  |  |  |  |  |  |  |  |  |  |  |  |  |  |  |  |
| 5. Doing housework W1 | 0.24 | 0.59 | 0.64 | 0.70 | - |  |  |  |  |  |  |  |  |  |  |  |  |  |  |  |  |  |
| 6. Taking medication W1 | 0.38 | 0.60 | 0.59 | 0.63 | 0.53 | - |  |  |  |  |  |  |  |  |  |  |  |  |  |  |  |  |
| 7. Handling finances W1 | 0.33 | 0.65 | 0.68 | 0.64 | 0.60 | 0.71 | - |  |  |  |  |  |  |  |  |  |  |  |  |  |  |  |
| 8. Sex W1 | -0.07 | -0.21 | -0.23 | -0.07 | -0.22 | -0.14 | -0.23 | - |  |  |  |  |  |  |  |  |  |  |  |  |  |  |
| 9. Age W1 | -0.11 | -0.25 | -0.30 | -0.32 | -0.36 | -0.26 | -0.35 | 0.11 | - |  |  |  |  |  |  |  |  |  |  |  |  |  |
| 10. SES W1 | 0.07 | 0.21 | 0.22 | 0.13 | 0.12 | 0.21 | 0.29 | -0.26 | -0.03 | - |  |  |  |  |  |  |  |  |  |  |  |  |
| 11. Length of residency W1 | 0.16 | 0.18 | 0.19 | 0.22 | 0.18 | 0.23 | 0.21 | -0.09 | -0.07 | 0.03 | - |  |  |  |  |  |  |  |  |  |  |  |
| 12. Partnership status W1 | 0.03 | 0.24 | 0.27 | 0.17 | 0.28 | 0.20 | 0.26 | -0.48 | -0.31 | 0.17 | 0.17 | - |  |  |  |  |  |  |  |  |  |  |
| 13. Living alone W1 | 0.14 | 0.08 | 0.04 | 0.22 | 0.02 | 0.16 | 0.10 | 0.33 | 0.11 | 0 | 0.04 | -0.68 | - |  |  |  |  |  |  |  |  |  |
| 14. Living in a nursing facility W1 | -0.20 | -0.35 | -0.36 | -0.46 | -0.33 | -0.46 | -0.43 | 0.15 | 0.35 | -0.09 | -0.45 | -0.22 | -0.21 | - |  |  |  |  |  |  |  |  |
| 15. At least weekly physically active W1 | 0.11 | 0.21 | 0.30 | 0.19 | 0.29 | 0.20 | 0.23 | -0.08 | -0.03 | 0.12 | 0.03 | 0.07 | 0.07 | -0.06 | - |  |  |  |  |  |  |  |
| 16. Neighborhood infrastructure ^b^ | -0.06 | -0.06 | -0.01 | -0.12 | -0.07 | -0.13 | -0.08 | -0.02 | 0.11 | -0.01 | -0.23 | -0.05 | -0.11 | 0.33 | 0.04 | - |  |  |  |  |  |  |
| 17. Neighborhood quality ^b^ | -0.05 | -0.01 | 0.06 | -0.04 | 0.04 | -0.03 | 0.01 | -0.06 | 0.06 | 0.08 | -0.05 | 0.07 | -0.12 | 0.17 | 0.09 | 0.61 | - |  |  |  |  |  |
| 18. Place attachment W1 | 0.09 | 0.10 | 0.15 | 0.16 | 0.15 | 0.17 | 0.15 | -0.04 | -0.06 | 0 | 0.32 | 0.08 | 0.04 | -0.17 | 0.07 | -0.03 | 0.14 | - |  |  |  |  |
| 19. Social cohesion W1 | 0.01 | 0.05 | 0.10 | 0.08 | 0.07 | 0.03 | 0.07 | -0.02 | -0.03 | -0.02 | 0.11 | 0.10 | -0.05 | 0.02 | 0.06 | 0.13 | 0.23 | 0.30 | - |  |  |  |
| 20. Walkability W1 | 0 | 0.04 | 0.10 | 0.02 | 0.06 | 0.02 | 0.07 | -0.06 | 0.01 | 0.08 | -0.20 | 0.02 | -0.06 | 0.14 | 0.09 | 0.36 | 0.13 | 0.06 | 0.12 | - |  |  |
| 21. Walkability difference ^c^ | 0.02 | 0.05 | 0.04 | 0 | -0.01 | 0.02 | 0.01 | 0.08 | 0.03 | -0.03 | 0.07 | -0.05 | 0.09 | -0.02 | -0.02 | -0.04 | 0 | -0.04 | -0.03 | -0.53 | - |  |
| 22. Place attachment difference ^c^ | -0.01 | 0.01 | -0.02 | -0.01 | -0.05 | -0.06 | -0.03 | 0.03 | 0.03 | -0.04 | 0.02 | -0.03 | 0.05 | -0.02 | -0.05 | -0.07 | -0.07 | -0.58 | -0.07 | -0.12 | 0.12 | - |
| 23. Social cohesion difference ^c^ | 0.06 | 0.05 | 0.04 | 0.06 | 0.07 | 0.05 | 0.07 | -0.01 | -0.07 | 0.09 | 0.03 | -0.06 | 0.08 | -0.13 | 0.07 | -0.06 | -0.02 | -0.08 | -0.51 | -0.06 | 0.04 | 0.11 |

*Note.* *N* = 840. Panel-weighted data. W1 = Wave 1; SES = Socioeconomic Status.

^a^ To simplify the illustration, only indicators of functional abilities at wave 1 are presented.

^b^ Mean value based on ratings from wave 1 and 2. Higher values indicate more favorable conditions.

^c^ Difference in ratings between wave 2 and 1. Negative values indicate a deterioration, and positive values indicate an improvement in rating.

**Supplementary Table 2.** *Cognitive Function: Correlations of Included Variables*

| Variables ^a^ | 1 | 2 | 3 | 4 | 5 | 6 | 7 | 8 | 9 | 10 | 11 | 12 | 13 | 14 | 15 | 16 | 17 | 18 | 19 | 20 |
| --- | --- | --- | --- | --- | --- | --- | --- | --- | --- | --- | --- | --- | --- | --- | --- | --- | --- | --- | --- | --- |
| 1. Immediate recall W1 | - |  |  |  |  |  |  |  |  |  |  |  |  |  |  |  |  |  |  |  |
| 2. Number transcoding W1 | 0.38 | - |  |  |  |  |  |  |  |  |  |  |  |  |  |  |  |  |  |  |
| 3. Word fluency W1 | 0.51 | 0.36 | - |  |  |  |  |  |  |  |  |  |  |  |  |  |  |  |  |  |
| 4. Digit span backwards W1 | 0.27 | 0.32 | 0.19 | - |  |  |  |  |  |  |  |  |  |  |  |  |  |  |  |  |
| 5. Delayed recall W1 | 0.50 | 0.30 | 0.40 | 0.21 | - |  |  |  |  |  |  |  |  |  |  |  |  |  |  |  |
| 6. Sex W1 | -0.02 | -0.12 | -0.12 | -0.01 | 0.03 | - |  |  |  |  |  |  |  |  |  |  |  |  |  |  |
| 7. Age W1 | -0.17 | -0.14 | -0.17 | -0.08 | -0.11 | 0.11 | - |  |  |  |  |  |  |  |  |  |  |  |  |  |
| 8. SES W1 | 0.17 | 0.28 | 0.15 | 0.19 | 0.19 | -0.26 | -0.01 | - |  |  |  |  |  |  |  |  |  |  |  |  |
| 9. Length of residency W1 | 0.19 | 0.21 | 0.18 | 0.07 | 0.20 | -0.09 | -0.06 | 0.02 | - |  |  |  |  |  |  |  |  |  |  |  |
| 10. Partnership status W1 | 0.10 | 0.09 | 0.10 | 0.08 | 0.07 | -0.48 | -0.30 | 0.16 | 0.16 | - |  |  |  |  |  |  |  |  |  |  |
| 11. Living alone W1 | 0.10 | 0.09 | 0.11 | 0.05 | 0.10 | 0.35 | 0.11 | -0.01 | 0.03 | -0.70 | - |  |  |  |  |  |  |  |  |  |
| 12. Living in a nursing facility W1 | -0.25 | -0.29 | -0.32 | -0.13 | -0.21 | 0.13 | 0.35 | -0.06 | -0.45 | -0.21 | -0.19 | - |  |  |  |  |  |  |  |  |
| 13. At least weekly physically active W1 | 0.11 | 0.13 | 0.09 | 0.04 | 0.11 | -0.08 | 0 | 0.13 | 0 | 0.05 | 0.05 | -0.04 | - |  |  |  |  |  |  |  |
| 14. Neighborhood infrastructure ^b^ | -0.04 | -0.08 | -0.10 | -0.03 | 0 | -0.03 | 0.11 | -0.01 | -0.22 | -0.05 | -0.10 | 0.33 | 0.07 | - |  |  |  |  |  |  |
| 15. Neighborhood quality ^b^ | 0 | 0.01 | -0.05 | 0.01 | 0.07 | -0.07 | 0.06 | 0.09 | -0.04 | 0.07 | -0.13 | 0.17 | 0.11 | 0.61 | - |  |  |  |  |  |
| 16. Place attachment W1 | 0.14 | 0.13 | 0.09 | 0.09 | 0.11 | -0.02 | -0.07 | 0.01 | 0.32 | 0.07 | 0.03 | -0.18 | 0.05 | -0.02 | 0.14 | - |  |  |  |  |
| 17. Social cohesion W1 | 0.02 | 0.01 | -0.01 | 0.01 | 0 | -0.03 | -0.02 | -0.02 | 0.08 | 0.10 | -0.09 | 0.03 | 0.03 | 0.16 | 0.24 | 0.28 | - |  |  |  |
| 18. Walkability W1 | -0.04 | -0.03 | -0.05 | -0.04 | -0.07 | -0.06 | 0.02 | 0.06 | -0.20 | 0.02 | -0.07 | 0.16 | 0.10 | 0.36 | 0.12 | 0.06 | 0.12 | - |  |  |
| 19. Walkability difference ^c^ | 0.03 | -0.02 | 0.05 | 0.03 | 0.01 | 0.08 | 0.02 | -0.02 | 0.07 | -0.05 | 0.10 | -0.02 | -0.03 | -0.03 | 0 | -0.05 | -0.02 | -0.52 | - |  |
| 20. Place attachment difference ^c^ | -0.05 | -0.07 | 0.02 | -0.05 | 0 | 0.02 | 0.05 | -0.05 | 0.02 | -0.02 | 0.04 | -0.02 | -0.07 | -0.07 | -0.07 | -0.59 | -0.09 | -0.13 | 0.13 | - |
| 21. Social cohesion difference ^c^ | 0.09 | 0.07 | 0.08 | 0.03 | 0.09 | 0 | -0.06 | 0.10 | 0.06 | -0.06 | 0.09 | -0.13 | 0.06 | -0.07 | -0.03 | -0.06 | -0.50 | -0.08 | 0.05 | 0.10 |

*Note.* *N* = 797. Panel-weighted data. W1 = Wave 1; SES = Socioeconomic Status.

^a^ To simplify the illustration, only indicators of cognitive function at wave 1 are presented.

^b^ Mean value based on ratings from wave 1 and 2. Higher values indicate more favorable conditions.

^c^ Difference in ratings between wave 2 and 1. Negative values indicate a deterioration and positive values indicate an improvement in rating.

**Supplementary Table 3.** *Comparison of Configural, Metric and Scalar Measurement Invariance for Cognitive Function and Functional Abilities Across the Two Waves*

| Degree of invariance | Cognitive Function (*n* = 797) | | | Functional Abilities (*n* = 840) | | |
| --- | --- | --- | --- | --- | --- | --- |
| (equality constraint) | Absolute model fit | Relative fit indices ^a^ | Absolute model fit | | Relative fit indices | Chi-square difference test ^b^ |
| Configural MI (factor model) | χ2 = 46.6, df = 29, *p* = .020 | RMSEA = 0.028, CFI = 0.985, SRM = 0.034 | χ2 = 346.6, df = 76, *p* < .001 | | RMSEA = 0.065, CFI = 0.984 |  |
| Metric MI (loadings) | χ2 = 52.4, df = 33, *p* = .017 | RMSEA = 0.027, CFI = 0.984, SRM = 0.042 | χ2 = 321.1, df = 82, *p* < .001 | | RMSEA = 0.059, CFI = 0.986 | Δχ2 = 9.2, Δdf = 6, *p* = .163 |
| Scalar MI (loadings and interceps/tresholds) | χ2 = 69.2, df = 37, *p* = .001 | RMSEA = 0.033, CFI = 0.973, SRM = 0.046 | χ2 = 348.1, df = 95, *p* < .001 | | RMSEA = 0.056, CFI = 0.985 | Δχ2 = 27.2, Δdf = 19, *p* = .100 |

*Note.* Panel-weighted data. MI = Measurement Invariance; RMSEA = Root Mean Square Error of Approximation; CFI = Comparative Fit Index; SRMR = Standardized Root Mean Square Residual.

^a^ The evaluation of model fit deterioration was based on Chen (2007): Significant deterioration of model fit in metric models is indicated by a CFI decline of minimum 0.010 accompanied by an increase in RMSEA of minimum 0.015 or an increase in SRMR of minimum 0.030; significant model fit deterioration in scalar models is determined by a CFI decline of minimum 0.010 accompanied with an increase in RMSEA of minimum 0.015 or an increase in SRMR of minimum 0.010.

^b^ Deterioration of model fit was evaluated using Chi-square difference test (Muthén & Muthén, 1998-2017).

**Supplementary Table 4.** *Results From the Latent Change Score Model for Changes in Functional Abilities Over the Two-Year Period*

|  |  | | Wave 1 | | | | | | | | | | Wave 2 | | | | | | | | | | | | | | | | | | | | | | |  |  |  |  |
| --- | --- | --- | --- | --- | --- | --- | --- | --- | --- | --- | --- | --- | --- | --- | --- | --- | --- | --- | --- | --- | --- | --- | --- | --- | --- | --- | --- | --- | --- | --- | --- | --- | --- | --- | --- | --- | --- | --- | --- |
|  |  | | *Estimate* | | | | *SE* | | *Std* | | | | *Estimate* | | | | | | | | *SE* | *Std* | | | | | | | | | | | | | |  |  |  |  |
| *Factor Loadings* | | |  | | | |  | |  | | | |  | | | | | | | | | | | | | | | | | | | | | | |  | |  |  |
| Using telephone | | | 1 | | | |  | | 0.886 | | | | 1 | | | | | | | |  | 0.985 | | | | | | | | | | | | | |  |  |  |  |
| Organizing routes | | | **1.225** | | | | 0.258 | | 0.919 | | | | **1.225** | | | | | | | | 0.258 | 1.032 | | | | | | | | | | | | | |  |  |  |  |
| Buying food and clothes | | | **1.466** | | | | 0.334 | | 0.941 | | | | **1.466** | | | | | | | | 0.334 | 1.064 | | | | | | | | | | | | | |  |  |  |  |
| Preparing meals | | | **1.365** | | | | 0.313 | | 0.933 | | | | **1.365** | | | | | | | | 0.313 | 1.053 | | | | | | | | | | | | | |  |  |  |  |
| Taking medication | | | **1.166** | | | | 0.226 | | 0.912 | | | | **1.166** | | | | | | | | 0.226 | 1.022 | | | | | | | | | | | | | |  |  |  |  |
| Doing housework | | | **1.004** | | | | 0.221 | | 0.886 | | | | **1.004** | | | | | | | | 0.221 | 0.986 | | | | | | | | | | | | | |  |  |  |  |
| Handling finances | | | **1.175** | | | | 0.254 | | 0.913 | | | | **1.175** | | | | | | | | 0.254 | 1.024 | | | | | | | | | | | | | |  |  |  |  |
|  | | |  | | | | | | | | | | | | | |  | | | | | | | | | | |  |  |  |  |  |  |  |  |  |  |  |  |
| *Thresholds (Reference: No help needed)* | | |  | | | | | | | | | | | | | |  | | | | | | | | | | |  |  |  |  |  |  |  |  |  |  |  |  |
| Using telephone | | |  | | | | | | | | | | | | | |  | | | | | | | | | | |  |  |  |  |  |  |  |  |  |  |  |  |
|  | Not possible without help | | **-4.202** | | | | 0.526 | |  | | | | **-4.202** | | | | | | | | 0.526 |  | | | | | | | | | | | | | |  |  |  |  |
|  | Some help needed | | **-3.589** | | | | 0.502 | |  | | | | **-3.589** | | | | | | | | 0.502 |  | | | | | | | | | | | | | |  |  |  |  |
| Organizing routes | | | | | |  | | | | | | | | | | | | | | | | | | | | | | | | | | | | |  |  |  |  |  |
|  | Not possible without help | | **-2.335** | | | | 0.190 | |  | | | | **-2.335** | | | | | | | | 0.190 |  | | | | | | | | | | | | | |  |  |  |  |
|  | Some help needed | | **-1.403** | | | | 0.175 | |  | | | | **-1.403** | | | | | | | | 0.175 |  | | | | | | | | | | | | | |  |  |  |  |
| Buying food and clothes | | | | | |  | | | | | | | | | | | | | | | | | | | | | | | | | | | | |  |  |  |  |  |
|  | Not possible without help | | **-2.397** | | | | 0.197 | |  | | | | **-2.397** | | | | | | | | 0.197 |  | | | | | | | | | | | | | |  |  |  |  |
|  | Some help needed | | **-1.214** | | | | 0.182 | |  | | | | **-1.214** | | | | | | | | 0.182 |  | | | | | | | | | | | | | |  |  |  |  |
| Preparing meals | | | | | |  | | | | | | | | | | | | | | | | | | | | | | | | | | | | |  |  |  |  |  |
|  | Not possible without help | | **-2.607** | | | | 0.245 | |  | | | | **-2.607** | | | | | | | | 0.245 |  | | | | | | | | | | | | | |  |  |  |  |
|  | Some help needed | | **-1.623** | | | | 0.216 | |  | | | | **-1.623** | | | | | | | | 0.216 |  | | | | | | | | | | | | | |  |  |  |  |
| Taking medication | | | | | |  | | | | | | | | | | | | | | | | | | | | | | | | | | | | |  |  |  |  |  |
|  | Not possible without help | | **-2.702** | | | | 0.237 | |  | | | | **-2.702** | | | | | | | | 0.237 | |  | | | |  |  |  |  |  |  |  |  |  |  |  |  |  |
|  | Some help needed | | **-1.991** | | | | 0.210 | |  | | | | **-1.991** | | | | | | | | 0.210 | |  | | | |  |  |  |  |  |  |  |  |  |  |  |  |  |
| Doing housework | | | | | |  | | | | | | | | | | | | | | | | | | | | | | | | | | | | |  |  |  |  |  |
|  | Not possible without help | | **-1.384** | | | | 0.128 | |  | | | | **-1.384** | | | | | | | | 0.128 | |  | | | |  |  |  |  |  |  |  |  |  |  |  |  |  |
|  | Some help needed | | **0.244** | | | | 0.114 | |  | | | | **0.244** | | | | | | | | 0.114 | |  | | | |  |  |  |  |  |  |  |  |  |  |  |  |  |
| Handling finances | | | | | |  | | | | | | | | | | | | | | | | | | | | | | | | | | | | |  |  |  |  |  |
|  | Not possible without help | | **-2.187** | | | | 0.164 | |  | | | | **-2.187** | | | | | | | | 0.164 | |  | | | |  |  |  |  |  |  |  |  |  |  |  |  |  |
|  | Some help needed | | **-1.173** | | | | 0.142 | |  | | | | **-1.173** | | | | | | | | 0.142 | |  | | | |  |  |  |  |  |  |  |  |  |  |  |  |  |
|  |  | |  | | | | | | |  | | | | | |  | | | | | | | | | |  | | | | |  |  |  |  |  |  |  |  |  |
| *Intercepts* | | |  | | | | | | | | | | | | | |  | | | | | | | | | | |  |  |  |  |  |  |  |  |  |  |  |  |
| Latent change score (functional abilities) | | | **-0.586** 0.131 | | | | | | | | | |  | | | | | | | |  | | | | | | | | |  |  |  |  |  |  |  |  |  |  |
|  |  | |  | | | |  | | | | | | |  | | | |  | | | | | | |  | | | | | | | | |  |  |  |  |  |  |
| *Regression* | | |  | | | | | | | | | | | | | |  | | | | | | |  | | | | | | | | |  |  |  |  |  |  |  |
| Functional abilities W1 🡪  Latent change score (functional abilities) | | | **-0.197** 0.042 -0.510 | | | | | | | | | |  | | | | | | | |  | | | | | | | |  | | | | | | | |  |  |  |
|  |  | |  |  | | | | | | |  | | | |  | | | |  | | |  | | | | | | | | | | | | |  |  |  |  |  |
| *Variances* | | |  | | | | | | | | | | | | | |  | | | | | | | | | | |  |  |  |  |  |  |  |  |  |  |  |  |
| Functional abilities W1 | | | **3.632** 1.547 | | | | | | | | | |  | | | | | | | |  | | | | | | | |  | | | | | | | |  |  |  |
| Latent change score (functional abilities) | | | **0.401** 0.168 | | | | | | | | | |  | | | | | | | |  | | | | | | | |  | | | | | | | |  |  |  |
| *Fit indices* | | | | | |  | | |  | | | |  | | | | | | | |  | |  | | | | | | | | | |  | | | | | | |
| RMSEA | | | 0.054 | | | | | |  | | | |  | | | | | | | |  | |  | | | | | | | | | |  | | | | | | |
| CFI | | | 0.984 | | | | | |  | | | |  | | | | | | | |  | |  | | | | | | | | | |  | | | | | | |

*Note.* *N* = 840. Panel-weighted data. SE = Standard Error; STD = Standardized Estimate; W1 = Wave 1; RMSEA = Root Mean Square Error of Approximation; CFI = Comparative Fit Index. Parameters which were statistically significant at the *p* < .05 level are given in bold font.

**Supplementary Table 5.** *Results From the Latent Change Score Model for Changes in Cognitive Function Over the Two-Year Period*

|  |  | | | Wave 1 | | | Wave 2 | | | | |
| --- | --- | --- | --- | --- | --- | --- | --- | --- | --- | --- | --- |
|  |  | | | Estimate | SE | Std | Estimate | SE | | | Std |
| *Factor Loadings* | | | |  |  |  |  |  | |  | |
| Immediate recall | | | | 1 |  | 0.715 | 1 |  | | 0.675 | |
| Number transcoding | | | | **0.713** | 0.076 | 0.533 | **0.713** | 0.076 | | 0.525 | |
| Word fluency | | | | **1.053** | 0.085 | 0.656 | **1.053** | 0.085 | | 0.599 | |
| Digit span backwards | | | | **0.439** | 0.047 | 0.453 | **0.439** | 0.047 | | 0.441 | |
| Delayed recall | | | | **1.822** | 0.162 | 0.606 | **1.822** | 0.162 | | 0.576 | |
|  |  |  |  |  |  |  |  | |  |  |  |
| *Intercepts* | | | |  |  |  |  |  | |  | |
| Immediate recall | | | | 0 | 0 |  | 0 | 0 | |  | |
| Number transcoding | | | | -0.049 | 0.041 |  | -0.049 | 0.041 | |  | |
| Word fluency | | | | -0.020 | 0.044 |  | -0.020 | 0.044 | |  | |
| Digit span backwards | | | | -0.019 | 0.024 |  | -0.019 | 0.024 | |  | |
| Delayed recall | | | | -0.127 | 0.071 |  | -0.127 | 0.071 | |  | |
| Latent change score (cognitive function) | | | | **-0.099** | 0.028 |  |  |  | |  | |
|  |  |  |  |  |  |  |  | |  |  |  |
| *Regression* | | | |  |  |  |  |  | |  | |
| Cognitive health W1 🡪  Latent change score (cognitive function) | | | | 0.009 | 0.070 | 0.018 |  |  | |  | |
|  |  |  |  |  |  |  |  | |  |  |  |
| *Covariances* | | | |  |  |  |  |  | |  | |
| Immediate recall over time | | | | **0.107** | 0.037 | 0.245 |  |  | |  | |
| Number transcoding over time | | | | **0.207** | 0.041 | 0.358 |  |  | |  | |
| Word fluency over time | | | | **0.257** | 0.054 | 0.350 |  |  | |  | |
| Digit span backwards over time | | | | **0.049** | 0.023 | 0.142 |  |  | |  | |
| Delayed recall over time | | | | **0.924** | 0.140 | 0.344 |  |  | |  | |
| Immediate and delayed recall W1 | | | | 0.126 | 0.068 | 0.116 |  |  | |  | |
| Immediate and delayed recall W2 | | | | 0.130 | 0.072 | 0.120 |  |  | |  | |
|  |  |  |  |  |  |  |  | |  |  |  |
| *Variances* | | | |  |  |  |  |  | |  | |
| Immediate recall | | | | **0.445** | 0.058 |  | **0.433** | 0.052 | |  | |
| Number transcoding | | | | **0.596** | 0.049 |  | **0.560** | 0.047 | |  | |
| Word fluency | | | | **0.685** | 0.066 |  | **0.788** | 0.068 | |  | |
| Digit span backwards | | | | **0.347** | 0.035 |  | **0.347** | 0.033 | |  | |
| Delayed recall | | | | **2.656** | 0.181 |  | **2.708** | 0.229 | |  | |
| Cognitive function W1 | | | | **0.465** | 0.088 |  |  |  | |  | |
| Latent change score (cognitive function) | | | | **0.114** | 0.041 |  |  |  | |  | |
| *Fit indices* | | | |  |  |  |  |  | |  | |
| RMSEA | | | | 0.027 |  |  |  |  | |  | |
| CFI | | | | 0.982 |  |  |  |  | |  | |
| SRMR | | | | 0.041 |  |  |  |  | |  | |

*Note.* *N* = 797. Panel-weighted data. SE = Standard Error; Std = Standardized Estimate; RMSEA = Root Mean Square Error of Approximation; CFI = Comparative Fit Index; SRMR = Standardized Root Mean Square Residual. Z-standardized values based on weighted means and standard deviations at baseline were used for manifest variables. Parameters which were statistically significant at the *p* < .05 level are given in bold font.

**Supplementary Table 6.** *Results From Latent Change Score Models Predicting Level and Change in Functional Abilities (Non-Imputed Data)*

|  | Functional Abilities: Model 1 (*n* = 809) | | | | | | Functional Abilities: Model 2 (*n* = 763) | | | | | |
| --- | --- | --- | --- | --- | --- | --- | --- | --- | --- | --- | --- | --- |
|  | Level |  |  | Change |  |  | Level |  |  | Change |  |  |
|  | Estimate | SE | Std | Estimate | SE | Std | Estimate | SE | Std | Estimate | SE | Std |
| Sex W1 (female) | **-0.550** | 0.256 | -0.086 | 0.386 | 0.209 | 0.129 | -0.524 | 0.281 | -0.086 | 0.343 | 0.217 | 0.121 |
| Age W1 | **-0.167** | 0.046 | -0.214 | -0.022 | 0.021 | -0.059 | **-0.156** | 0.047 | -0.207 | -0.030 | 0.022 | -0.086 |
| SES W1 | **0.019** | 0.007 | 0.125 | 0.001 | 0.004 | 0.016 | **0.020** | 0.008 | 0.141 | 0 | 0.005 | -0.004 |
| Length of residency W1 | 0.010 | 0.005 | 0.082 | 0.004 | 0.003 | 0.078 | 0.002 | 0.005 | 0.017 | 0.008 | 0.004 | 0.153 |
| Married/partnered W1 | **1.914** | 0.618 | 0.305 | -0.587 | 0.309 | -0.199 | **1.457** | 0.539 | 0.242 | -0.377 | 0.321 | -0.135 |
| Living alone W1 | **1.849** | 0.544 | 0.297 | -0.364 | 0.231 | -0.125 | **1.393** | 0.482 | 0.233 | -0.159 | 0.273 | -0.058 |
| Living in a nursing care facility W1 | **-2.071** | 0.714 | -0.202 | **1.062** | 0.498 | 0.221 | **-2.440** | 0.770 | -0.240 | **1.175** | 0.519 | 0.250 |
| At least weekly physically active W1 | **1.729** | 0.460 | 0.200 | **-0.499** | 0.244 | -0.123 | **1.794** | 0.554 | 0.214 | -0.393 | 0.230 | -0.101 |
| Neighborhood infrastructure ^a^ | 0.008 | 0.253 | 0.002 | -0.132 | 0.171 | -0.058 | 0.031 | 0.253 | 0.007 | -0.143 | 0.176 | -0.066 |
| Neighborhood quality ^a^ | 0.218 | 0.235 | 0.045 | -0.048 | 0.153 | -0.021 | 0.064 | 0.242 | 0.014 | -0.033 | 0.153 | -0.016 |
| Walkability W1 | **0.457** | 0.184 | 0.145 | -0.058 | 0.111 | -0.039 | **0.385** | 0.176 | 0.128 | 0.003 | 0.107 | 0.002 |
| Walkability difference ^b^ |  |  |  | **0.347** | 0.125 | 0.246 |  |  |  | **0.323** | 0.132 | 0.242 |
| Place attachment W1 |  |  |  |  |  |  | 0.208 | 0.175 | 0.066 | -0.158 | 0.163 | -0.108 |
| Social cohesion W1 |  |  |  |  |  |  | 0.066 | 0.145 | 0.022 | 0.057 | 0.120 | 0.042 |
| Place attachment difference ^b^ |  |  |  |  |  |  |  |  |  | 0.003 | 0.103 | 0.002 |
| Social cohesion difference ^b^ |  |  |  |  |  |  |  |  |  | 0.156 | 0.114 | 0.109 |
| R-Square | 0.431 |  |  | 0.209 |  |  | 0.403 |  |  | 0.204 |  |  |
| RMSEA | 0.035 |  |  |  |  |  | 0.028 |  |  |  |  |  |
| CFI | 0.971 |  |  |  |  |  | 0.976 |  |  |  |  |  |

*Note.* Panel-weighted data. SE = Standard Error; Std = Standardized Estimate; W1 = Wave 1; W2 = Wave 2; SES = Socioeconomic Status; RMSEA = Root Mean Square Error of Approximation; CFI = Comparative Fit Index. Parameters which were statistically significant at the *p* < .05 level are given in bold font.

^a^ Mean value based on the ratings of wave 1 and 2. Higher values indicate more favorable conditions.

^b^ Difference in ratings between wave 2 and 1. Negative values indicate a deterioration and positive values indicate an improvement in rating.

**Supplementary Table 7.** *Results From Latent Change Score Models Predicting Level and Change in Cognitive Function (Non-Imputed Data)*

|  | Cognitive Function: Model 1 (*n* = 769) | | | | | | Cognitive Function: Model 2 (*n* = 726) | | | | | |
| --- | --- | --- | --- | --- | --- | --- | --- | --- | --- | --- | --- | --- |
|  | Level |  |  | Change |  |  | Level |  |  | Change | | |
|  | Estimate | SE | Std | Estimate | SE | Std | Estimate | SE | Std | Estimate | SE | Std |
| Sex W1 (female) | **0.181** | 0.064 | 0.136 | 0.035 | 0.040 | 0.051 | **0.164** | 0.061 | 0.129 | 0.032 | 0.042 | 0.045 |
| Age W1 | **-0.015** | 0.003 | -0.092 | **-0.005** | 0.002 | -0.062 | **-0.015** | 0.004 | -0.092 | **-0.006** | 0.002 | -0.069 |
| SES W1 | **0.009** | 0.002 | 0.295 | 0 | 0.001 | -0.017 | **0.009** | 0.002 | 0.299 | 0 | 0.001 | -0.020 |
| Length of residency W1 | **0.004** | 0.001 | 0.156 | 0.002 | 0.001 | 0.150 | **0.003** | 0.001 | 0.126 | 0.002 | 0.001 | 0.140 |
| Married/partnered W1 | **0.265** | 0.119 | 0.202 | **0.182** | 0.078 | 0.272 | **0.255** | 0.113 | 0.203 | **0.182** | 0.084 | 0.263 |
| Living alone W1 | **0.263** | 0.112 | 0.203 | 0.128 | 0.093 | 0.192 | **0.263** | 0.106 | 0.211 | 0.122 | 0.101 | 0.177 |
| Living in a nursing facility W1 | **-0.480** | 0.217 | -0.219 | 0.249 | 0.156 | 0.221 | **-0.545** | 0.221 | -0.249 | 0.254 | 0.168 | 0.211 |
| At least weekly physically active W1 | 0.182 | 0.103 | 0.099 | -0.014 | 0.077 | -0.015 | 0.203 | 0.106 | 0.115 | -0.009 | 0.081 | -0.009 |
| Neighborhood infrastructure ^a^ | 0.087 | 0.067 | 0.086 | 0.003 | 0.059 | 0.006 | 0.120 | 0.065 | 0.124 | -0.015 | 0.060 | -0.028 |
| Neighborhood quality ^a^ | -0.015 | 0.059 | -0.015 | 0.007 | 0.050 | 0.014 | -0.022 | 0.064 | -0.023 | -0.010 | 0.052 | -0.020 |
| Walkability W1 | -0.023 | 0.031 | -0.036 | 0.014 | 0.030 | 0.041 | -0.041 | 0.032 | -0.065 | 0.010 | 0.031 | 0.028 |
| Walkability difference ^b^ |  |  |  | 0.003 | 0.027 | 0.009 |  |  |  | -0.004 | 0.027 | -0.011 |
| Place attachment W1 |  |  |  |  |  |  | 0.057 | 0.043 | 0.086 | -0.045 | 0.030 | -0.122 |
| Social cohesion W1 |  |  |  |  |  |  | -0.043 | 0.028 | -0.068 | **0.081** | 0.034 | 0.232 |
| Place attachment difference ^b^ |  |  |  |  |  |  |  |  |  | -0.007 | 0.023 | -0.022 |
| Social cohesion difference ^b^ |  |  |  |  |  |  |  |  |  | 0.025 | 0.028 | 0.069 |
| R-Square | 0.298 |  |  | 0.053 |  |  | 0.326 |  |  | 0.089 |  |  |
| RMSEA | 0.032 |  |  |  |  |  | 0.029 |  |  |  |  |  |
| CFI | 0.934 |  |  |  |  |  | 0.935 |  |  |  |  |  |
| SRMR | 0.044 |  |  |  |  |  | 0.042 |  |  |  |  |  |

*Note.* Panel-weighted data. SE = Standard Error; Std = Standardized Estimate; W1 = Wave 1; SES = Socioeconomic Status; RMSEA = Root Mean Square Error of Approximation; CFI = Comparative Fit Index; SRMR = Standardized Root Mean Square Residual. Z-standardized values based on weighted means and standard deviations at baseline were used for manifest variables. Parameters which were statistically significant at the *p* < .05 level are given in bold font.

^a^ Mean value based on the ratings of wave 1 and 2. Higher values indicate more favorable conditions.

^b^ Difference in ratings between wave 2 and 1. Negative values indicate a deterioration and positive values indicate an improvement in rating.

**Supplementary Table 8.** *Results of Path Analysis Examining Mediational Pathways from Physical Neighborhood Characteristics to Level and Change in Functional Abilities of the Oldest Old*

|  |  | Level of functional abilities | | | | | | | | | | | | | | | |
| --- | --- | --- | --- | --- | --- | --- | --- | --- | --- | --- | --- | --- | --- | --- | --- | --- | --- |
|  |  | Direct effect | | |  | Total indirect effect | | |  | Specific indirect effect | | |  | Total effect | | |  |
| Exogenous variable | Mediator | Est | SE | Std |  | Est | SE | Std |  | Est | SE | Std |  | Est | SE | Std |  |
| Neighborhood infrastructure ^a^ |  | 0.118 | 0.182 | 0.034 |  | -0.020 | 0.019 | -0.006 |  |  |  |  |  | 0.099 | 0.180 | 0.028 |  |
|  | Place attachment W1 |  |  |  |  |  |  |  |  | -0.017 | 0.018 | -0.005 |  |  |  |  |  |
|  | Social cohesion W1 |  |  |  |  |  |  |  |  | -0.002 | 0.006 | -0.001 |  |  |  |  |  |
| Neighborhood quality ^a^ |  | 0.083 | 0.182 | 0.024 |  | 0.047 | 0.038 | 0.013 |  |  |  |  |  | 0.130 | 0.172 | 0.037 |  |
|  | Place attachment W1 |  |  |  |  |  |  |  |  | 0.031 | 0.027 | 0.009 |  |  |  |  |  |
|  | Social cohesion W1 |  |  |  |  |  |  |  |  | 0.017 | 0.029 | 0.005 |  |  |  |  |  |
| Walkability W1 |  | **0.191** | 0.095 | 0.082 |  | 0.020 | 0.016 | 0.008 |  |  |  |  |  | **0.211** | 0.094 | 0.091 |  |
|  | Place attachment W1 |  |  |  |  |  |  |  |  | 0.013 | 0.012 | 0.006 |  |  |  |  |  |
|  | Social cohesion W1 |  |  |  |  |  |  |  |  | 0.006 | 0.012 | 0.003 |  |  |  |  |  |
|  |  |  |  |  |  |  |  |  |  |  |  |  |  |  |  |  |  |
|  |  | Change in functional abilities | | | | | | | | | | | | | | | |
|  |  | Direct effect | | |  | Total indirect effect | | |  | Specific indirect effect | | |  | Total effect | | |  |
| Exogenous variable | Mediator | Est | SE | Std |  | Est | SE | Std |  | Est | SE | Std |  | Est | SE | Std |  |
| Neighborhood infrastructure ^a^ |  | -0.090 | 0.114 | -0.059 |  | 0.011 | 0.013 | 0.007 |  |  |  |  |  | -0.079 | 0.117 | -0.052 |  |
|  | Place attachment W1 |  |  |  |  |  |  |  |  | 0.012 | 0.014 | 0.008 |  |  |  |  |  |
|  | Social cohesion W1 |  |  |  |  |  |  |  |  | 0 | 0.004 | 0 |  |  |  |  |  |
| Neighborhood quality ^a^ |  | 0.019 | 0.113 | 0.012 |  | -0.017 | 0.026 | -0.011 |  |  |  |  |  | 0.001 | 0.111 | 0.001 |  |
|  | Place attachment W1 |  |  |  |  |  |  |  |  | -0.020 | 0.023 | -0.013 |  |  |  |  |  |
|  | Social cohesion W1 |  |  |  |  |  |  |  |  | 0.003 | 0.024 | 0.002 |  |  |  |  |  |
| Walkability W1 |  | -0.062 | 0.062 | -0.062 |  | -0.008 | 0.011 | -0.008 |  |  |  |  |  | -0.070 | 0.063 | -0.070 |  |
|  | Place attachment W1 |  |  |  |  |  |  |  |  | -0.009 | 0.009 | -0.009 |  |  |  |  |  |
|  | Social cohesion W1 |  |  |  |  |  |  |  |  | 0.001 | 0.009 | 0.001 |  |  |  |  |  |
|  |  |  |  |  |  |  |  |  |  |  |  |  |  |  |  |  |  |
| RMSEA |  | 0.033 |  |  |  |  |  |  |  |  |  |  |  |  |  |  |  |
| CFI |  | 0.971 |  |  |  |  |  |  |  |  |  |  |  |  |  |  |  |

*Note.* *N* = 840. Panel-weighted data. Est = Unstandardized Estimate; SE = Standard Error; Std = Standardized Estimate; W1 = Wave 1; SES = Socioeconomic Status; RMSEA = Root Mean Square Error of Approximation; CFI = Comparative Fit Index. Following variables were included as confounders of both mediators and level/change in functional abilities: age, sex, partnership status, living alone, length of residency, living in a nursing care facility, individual socioeconomic status, and physical activity. Parameters which were statistically significant at the *p* < .05 level are given in bold font.

^a^ Mean value based on the ratings of wave 1 and 2. Higher values indicate more favorable conditions.

**Supplementary Table 9.** *Results of Path Analysis Examining Mediational Pathways from Physical Neighborhood Characteristics to Level and Change in Cognitive Function of the Oldest Old*

|  |  | Level of cognitive function | | | | | | | | | | | | | | | |
| --- | --- | --- | --- | --- | --- | --- | --- | --- | --- | --- | --- | --- | --- | --- | --- | --- | --- |
|  |  | Direct effect | | |  | Total indirect effect | | |  | Specific indirect effect | | |  | Total effect | | |  |
| Exogenous variable | Mediator | Est | SE | Std |  | Est | SE | Std |  | Est | SE | Std |  | Est | SE | Std |  |
| Neighborhood infrastructure ^a^ |  | 0.100 | 0.065 | 0.099 |  | -0.008 | 0.008 | -0.008 |  |  |  |  |  | 0.091 | 0.064 | 0.090 |  |
|  | Place attachment W1 |  |  |  |  |  |  |  |  | -0.008 | 0.008 | -0.008 |  |  |  |  |  |
|  | Social cohesion W1 |  |  |  |  |  |  |  |  | 0 | 0.001 | 0 |  |  |  |  |  |
| Neighborhood quality ^a^ |  | -0.023 | 0.065 | -0.023 |  | 0.012 | 0.016 | 0.012 |  |  |  |  |  | -0.012 | 0.060 | -0.011 |  |
|  | Place attachment W1 |  |  |  |  |  |  |  |  | 0.017 | 0.012 | 0.017 |  |  |  |  |  |
|  | Social cohesion W1 |  |  |  |  |  |  |  |  | -0.005 | 0.010 | -0.005 |  |  |  |  |  |
| Walkability W1 |  | -0.037 | 0.032 | -0.055 |  | 0.006 | 0.006 | 0.009 |  |  |  |  |  | -0.031 | 0.031 | -0.046 |  |
|  | Place attachment W1 |  |  |  |  |  |  |  |  | 0.007 | 0.005 | 0.011 |  |  |  |  |  |
|  | Social cohesion W1 |  |  |  |  |  |  |  |  | -0.002 | 0.003 | -0.002 |  |  |  |  |  |
|  |  |  |  |  |  |  |  |  |  |  |  |  |  |  |  |  |  |
|  |  | Change in cognitive function | | | | | | | | | | | | | | | |
|  |  | Direct effect | | |  | Total indirect effect | | |  | Specific indirect effect | | |  | Total effect | | |  |
| Exogenous variable | Mediator | Est | SE | Std |  | Est | SE | Std |  | Est | SE | Std |  | Est | SE | Std |  |
| Neighborhood infrastructure ^a^ |  | -0.022 | 0.058 | -0.043 |  | 0.004 | 0.007 | 0.009 |  |  |  |  |  | -0.017 | 0.058 | -0.035 |  |
|  | Place attachment W1 |  |  |  |  |  |  |  |  | 0.004 | 0.005 | 0.008 |  |  |  |  |  |
|  | Social cohesion W1 |  |  |  |  |  |  |  |  | 0 | 0.005 | 0.001 |  |  |  |  |  |
| Neighborhood quality ^a^ |  | -0.015 | 0.051 | -0.031 |  | 0.014 | 0.012 | 0.029 |  |  |  |  |  | -0.001 | 0.050 | -0.002 |  |
|  | Place attachment W1 |  |  |  |  |  |  |  |  | -0.008 | 0.009 | -0.016 |  |  |  |  |  |
|  | Social cohesion W1 |  |  |  |  |  |  |  |  | **0.022** | 0.011 | 0.045 |  |  |  |  |  |
| Walkability W1 |  | 0.008 | 0.026 | 0.025 |  | 0.004 | 0.005 | 0.012 |  |  |  |  |  | 0.012 | 0.026 | 0.037 |  |
|  | Place attachment W1 |  |  |  |  |  |  |  |  | -0.003 | 0.004 | -0.011 |  |  |  |  |  |
|  | Social cohesion W1 |  |  |  |  |  |  |  |  | 0.007 | 0.005 | 0.023 |  |  |  |  |  |
|  |  |  |  |  |  |  |  |  |  |  |  |  |  |  |  |  |  |
| RMSEA |  | 0.028 |  |  |  |  |  |  |  |  |  |  |  |  |  |  |  |
| CFI |  | 0.951 |  |  |  |  |  |  |  |  |  |  |  |  |  |  |  |
| SRMR |  | 0.040 |  |  |  |  |  |  |  |  |  |  |  |  |  |  |  |

*Note.* *N* = 797. Panel-weighted data. Est = Unstandardized Estimate; SE = Standard Error; Std = Standardized Estimate; W1 = Wave 1; SES = Socioeconomic Status; RMSEA = Root Mean Square Error of Approximation; CFI = Comparative Fit Index; SRMR = Standardized Root Mean Square Residual. Z-standardized values based on weighted means and standard deviations at baseline were used for manifest variables. Following variables were included as confounders of both mediators and level/change in functional abilities: age, sex, partnership status, living alone, length of residency, living in a nursing care facility, individual socioeconomic status, and physical activity. Parameters that were statistically significant at the *p* < .05 level are given in bold font.

^a^ Mean value based on ratings of wave 1 and 2. Higher values indicate more favorable conditions.

**References**

Chen, F. F. (2007). Sensitivity of goodness of fit indexes to lack of measurement invariance. *Structural Equation Modeling: A Multidisciplinary Journal*(3), 464–504. https://doi.org/10.1080/1070551070

Muthén, L. K., & Muthén, B. O. (1998-2017). *Mplus User's Guide* (Eighth Edition). Muthén & Muthén.
